# Nonalcoholic fatty liver disease developed after pancreatoduodenectomy for solid pseudopapillary neoplasm in a 10-year-old girl: a case report

**DOI:** 10.1186/s40792-022-01414-9

**Published:** 2022-04-06

**Authors:** Toshio Sawai, Shogo Zuo, Taichi Terai, Satoshi Nishiwada, Kenji Nakagawa, Minako Nagai, Takehiro Akahori, Hiromichi Kanehiro, Masayuki Sho

**Affiliations:** Department of Surgery, Nara Medical University, 840 Shijo-chou, Kashihara, Nara 634-8522 Japan

**Keywords:** Solid pseudopapillary neoplasm, Pancreatoduodenectomy, Postoperative complications, Nonalcoholic fatty liver disease, Supplementation therapy of exocrine pancreatic enzymes, Child

## Abstract

**Background:**

Solid pseudopapillary neoplasms of the pancreas are rare. Moreover, pancreatoduodenectomy (PD) and postoperative care are not common in pediatric surgery. Herein, we report a case of PD and nonalcoholic fatty liver disease (NAFLD) after PD and present a literature review.

**Case presentation:**

A 10-year-old girl with a suspected liver tumor was referred to our hospital. Echography, enhanced computed tomography and magnetic resonance imaging showed that the tumor coexisted with the solid and cystic parts of the pancreatic head. Since the patient was a young woman and the imaging findings were consistent with that of pancreatic solid pseudopapillary neoplasms (SPNs), we diagnosed her with pancreatic SPN. Thereafter, PD was performed, and she was discharged 10 days after the operation. Although her postoperative course was mostly uneventful, she experienced few episodes of abdominal pain and diarrhea before hospital discharge. These symptoms subsequently became more frequent and severe. The patient was urgently readmitted to the hospital for watery steatorrhea and lower abdominal colic pain. Her serum aspartate aminotransferase and alanine aminotransferase levels were elevated, and a fatty liver was detected on echography. The patient was diagnosed with steatorrhea, peristaltic pain, and NAFLD after PD. Pancrelipase (containing pancreatic digestive enzymes), antidiarrheal agents, and probiotics were started. Dosage increase of these drugs reduced the defecation frequency and abdominal pain and switched diarrhea to loose stools. However, more lipids in meals or more meals caused diarrhea and abdominal pain. Therefore, the doses of these drugs were further increased, and another antidiarrheal agent, loperamide hydrochloride, was added. Exocrine pancreatic enzymes supplementation and careful follow-up should prevent NAFLD progression after PD. At present, the patient has occasional abdominal pain, but has tangible soft stools once or twice a day. Although echography still shows a mottled fatty liver, her hepatic enzymes are only mildly elevated.

**Conclusions:**

Pediatric PD is rare, and residual pancreatic function is usually sufficient, unlike in adult cases. However, we experienced a case of NAFLD after PD for a pediatric pancreatic SPN, in which pancreatic enzyme supplementation effectively improved this condition. Further attention must be paid to worsening of NAFLD that can develop nonalcoholic steatohepatitis.

## Background

Pancreatic solid pseudopapillary neoplasms (SPNs) are rare tumors accounting for approximately 1–3% of all pancreatic tumors and occur most commonly in the pancreatic body or tail in young women. Surgical resection results in good prognosis of pancreatic SPNs. However, local or distant recurrence has occasionally been reported. Therefore, SPNs are considered as low grade malignant tumors [1]. In adults, pancreatoduodenectomy (PD) is known to result in a high rate of nonalcoholic fatty liver disease (NAFLD) as a postoperative complication, leading to fatty liver disease due to pancreatectomy and residual pancreatic hypofunction.

Herein, we report a case of PD for a large SPN in the pancreatic head and the development of NAFLD in a 10-year-old girl, and present a literature review.

## Case presentation

The patient was a 10-year-old girl. Two months before admission, she experienced fullness in the upper right abdomen but could eat without nausea or vomiting. However, she has had a small meal since before and usually takes more than an hour to eat. When she complained of these signs to a former physician at the time of influenza vaccination, a liver tumor was suspected on echography and was referred to our department for an evaluation.

On admission, the patient’s height, weight and body mass index were 140 cm, 28.7 kg, and 14.6, respectively. The upper right abdomen was swollen and a mass was palpable. This mass was 10 cm in size, elastic, and hard with a clear border and no mobility. Blood tests on admission showed no increases in biliary enzymes, deviant liver enzymes, or tumor markers.

**Fig. 1:**
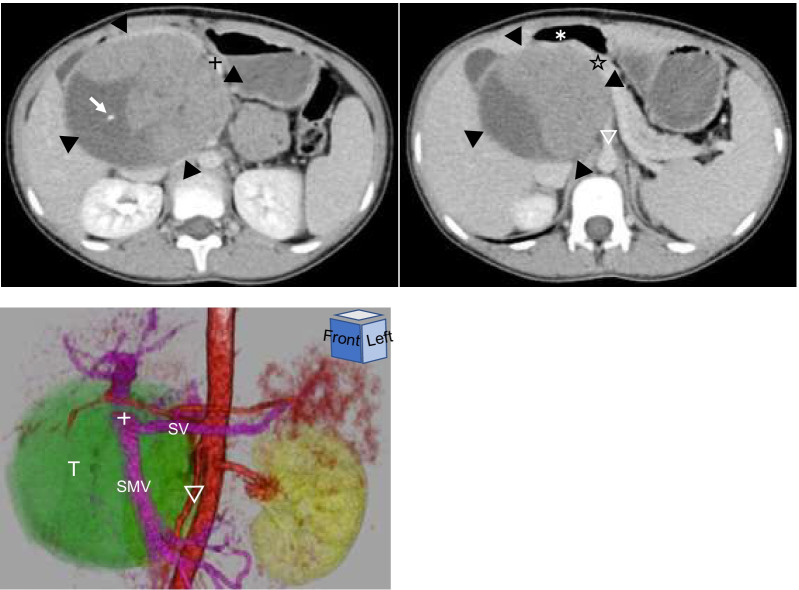
**Contrast-enhanced CT scan and 3D imaging on admission.** The tumor is located in the retroperitoneum, including the pancreatic head, in such a way that the pancreas (☆) is ventralized, the duodenum (✻) is right ventralized, and the extrahepatic portal vein (+) is left ventralized. The mass is well-defined, smooth, and covered with a thick coat-like structure (surrounded by ▼), with thin septum-like structures with microcalcification (→) and papillary enhancing components within the tumor. In the 3D image from the left anterior view, the extrahepatic portal vein (+) runs ventrally to the left in contact with the tumor surface, and the SMA (▽) runs to the left in contact with the tumor surface. *CT* computed tomography, *SMV* superior mesenteric vein, *SV* splenic vein, *T* tumor

Echography on admission revealed that the mass was not related to the liver but was continuous with the pancreatic head. The inferior vena cava was pushed to the dorsal side, the portal vein was located on the left side, and the duodenum was shown on the right and ventral sides. The mass boundary was clear. No fatty liver or other liver abnormalities were observed. Contrast-enhanced computed tomography (CT) examination on admission showed a large tumor (94 × 85 × 75 mm) with a well-defined, smooth, thick capsular structure arising from the pancreatic head. Moreover, a thin septal structure with microcalcification was found in the tumor. Cystic and solid papillary components were observed (Fig. 1). Magnetic resonance imaging (MRI) on admission revealed tumor with a diameter of 10 cm in the pancreatic head.

**Fig. 2:**
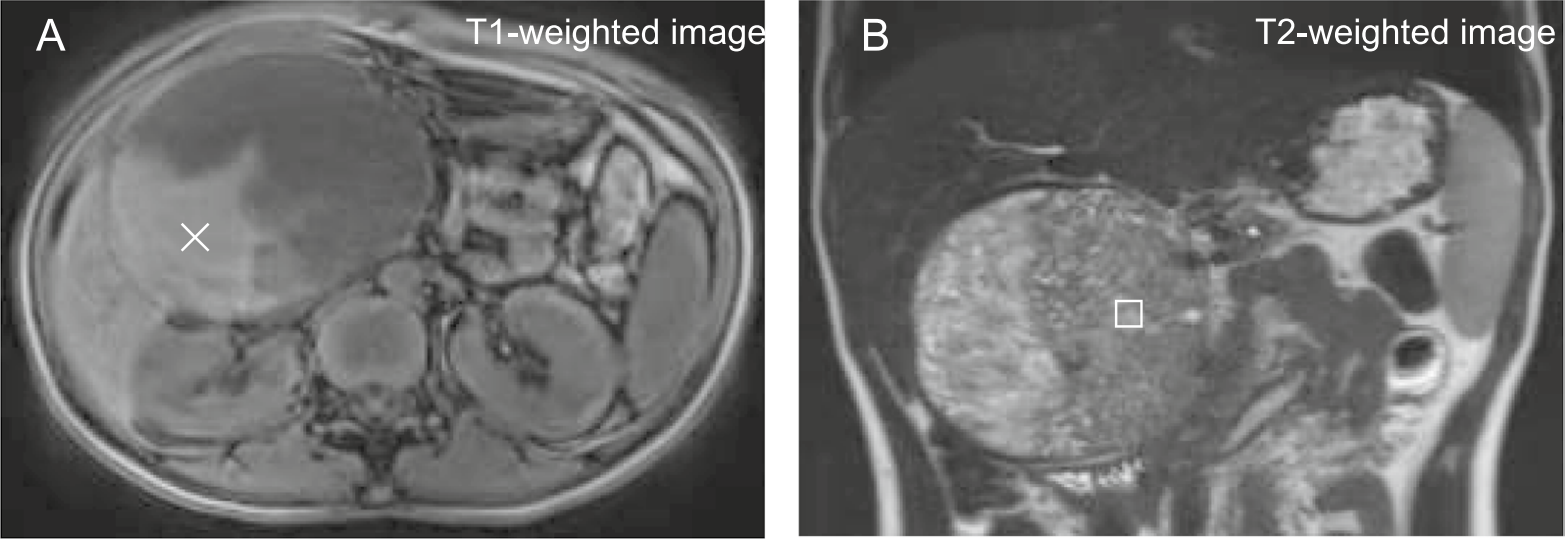
**MRI on admission.** The tumor’s internal structure is heterogeneous, and the T1-weighted image (**A**) shows a high signal (×), suggesting hemorrhagic changes. In the enhanced area, the T2-weighted image (**B**) shows predominantly intermediate signals (□), with a mixture of vesicular and slit-like high-signal areas. Therefore, the findings show a mixture of a substantial mass and hemorrhagic and degenerative necrotic areas. MRI, magnetic resonance imaging

Inside the tumor, T1-weighted imaging showed a high signal. In addition, in the solid part, T2-weighted imaging primarily showed an intermediate signal, with small cyst-like and slit-like hyperintensities mixed within, indicating that the solid and hemorrhagic degenerative necrotic components were mixed (Fig. 2). Bone scintigraphy on admission revealed no bone metastases.

A preoperative diagnosis of SPN in the pancreatic head was established. We planned to attempt enucleation at first, and possibly convert to PD, if enucleation was difficult.

**Fig. 3:**
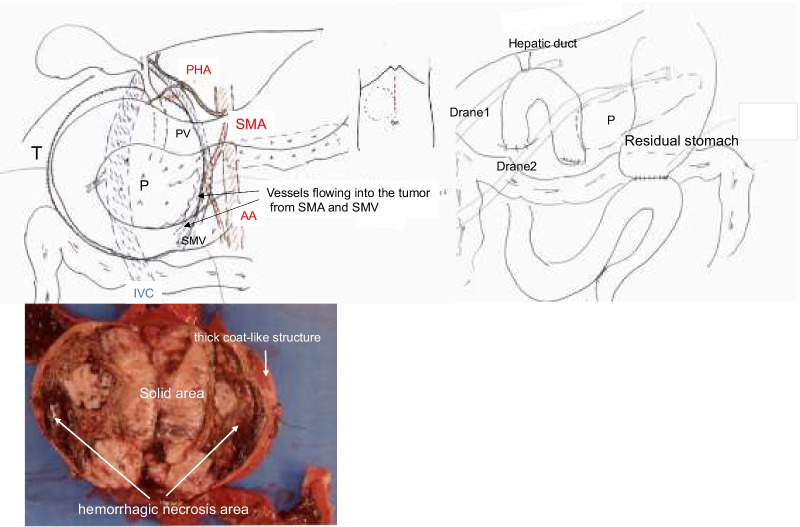
**Surgical findings and resection specimen sections.** The abdomen is opened through a midline incision on the upper abdomen. The tumor (T) is located on the dorsal side of the pancreatic (P) head, which is ventrally compressed and thinned. The superior mesenteric vein (SMV), extrahepatic portal vein (PV), and SMA are highly adherent to the tumor but can be dissected while ligating the arteriovenous branches to the tumor. Reconstruction consists of anastomosis of the upper intestine, pancreas, bile duct, and stomach. Braun anastomosis is performed. Drains are placed dorsal (drain 1) and ventral (drain 2) to the bile duct jejunal anastomosis and pancreatic jejunal anastomosis, respectively. Tumor section: The tumor is covered with a thick coat-like structure, and the interior is a mixture of substantial and hemorrhagic necrotic areas. *IVC* inferior vena cava, *AA* abdominal aorta, *SMA* superior mesenteric artery

Under general anesthesia, laparotomy (Fig. 3) was performed using a midline incision in the upper abdomen. The tumor was located on the dorsal side of the duodenum and pancreatic head. A small amount of ascites was found and submitted for cytopathology, and no tumor cells were detected. First, the duodenum and colon were detached from the tumor wall for enucleation. The parenchyma of the pancreatic head was confirmed, and the tumor was detached. Adhesions became severe in the vicinity of the pancreatic uncinate, and peeling was difficult, resulting in a cut into the pancreatic parenchyma. This part was inferred as the primary site, and postoperative pancreatic juice leakage was a strong concern. Therefore, enucleation was abandoned, and PD was performed. Although the tumor was strongly adhered to the superior mesenteric artery (SMA), it was removed. However, partial removal of the SMA plexus was necessary to remove the tumor without damaging it. In addition to subtotal stomach-preserving PD and modified Child reconstruction, Braun anastomosis was added. Pancreatojejunostomy was performed using pancreatojejunal mucosal anastomosis and the modified Blumgart method.

Examining the cut surface of the resected specimen confirmed that a thick capsule-like structure surrounded it and solid and hemorrhagic degenerative necrotic parts were mixed inside. The pathological findings confirmed a diagnosis of pancreatic SPNs.

**Fig. 4:**
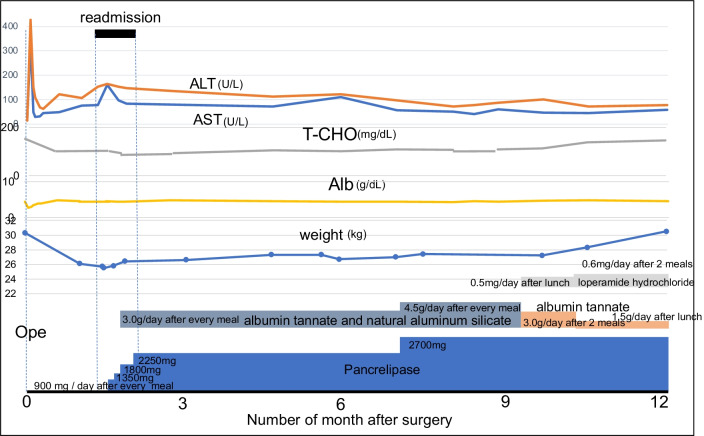
**Postoperative course.** The postoperative course from immediately before surgery to the present is shown. The top row shows AST/ALT levels, total cholesterol, albumin, and weight. The bottom row shows medications that are used. The AST/ALT levels are elevated again at readmission after recovery from acute postoperative elevation. It has progressively reduced with the diagnosis and initiation of treatment for NAFLD. The total cholesterol level decreased after the surgery, and after the lowest value of 94 mg/dL at the time of readmission. It was restored to the normal range one year after the surgery following the diagnosis of NAFLD and initiation of treatment. Albumin levels remain unchanged, except for in the acute postoperative period. The patient’s weight is 30.3 kg immediately preoperatively, but decreased to 25.4 kg on readmission. Subsequently, with the diagnosis of NAFLD and initiation of treatment, she recovered to her preoperative weight. *ALT* alanine aminotransferase, *NAFLD* nonalcoholic fatty liver disease, *AST* aspartate aminotransferase

The postoperative course is shown in Fig. 4. Oral water intake was started one day after the operation, and a fat-restricted diet was started 3 days postoperatively. No pancreatic juice leakage was observed. However, diarrhea and abdominal pain occurred on postoperative day four. The patient was discharged on postoperative day 10 because her symptoms gradually improved. Even after discharge, she had loose stools approximately three times a day. Furthermore, upon consumption of fatty food, she had diarrhea again and her abdominal pain worsened.

**Fig. 5:**
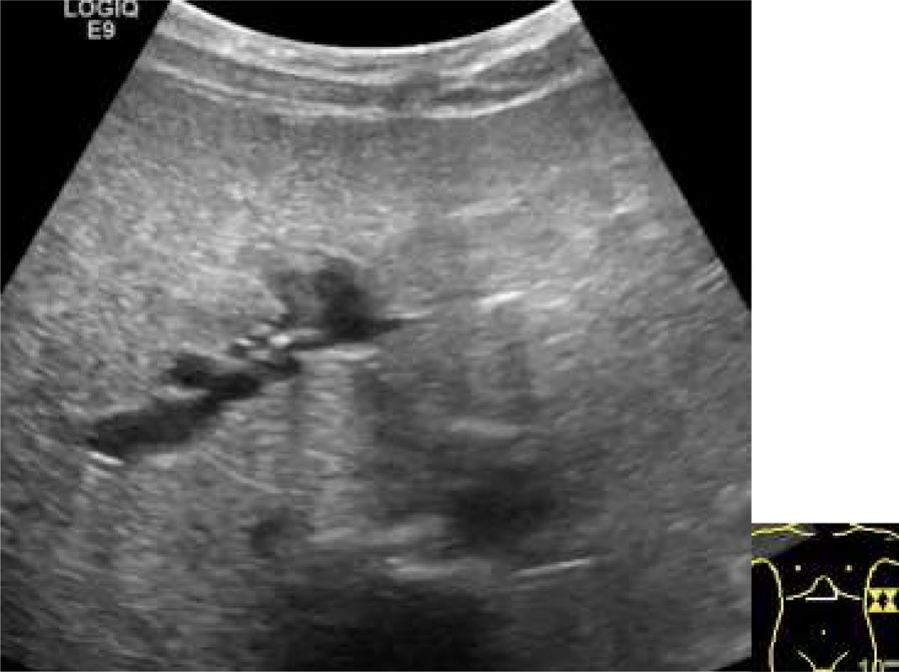
**Echography on readmission.** Abdominal echography on readmission shows a spotted high echoic pattern in liver

Immediately postoperatively, blood tests showed increases in serum aspartate aminotransferase (AST, 342 U/L) and alanine aminotransferase (ALT, 428 U/L), which were thought to be due to the operation. However, serum concentration of AST and ALT decreased one week after the operation to 31 U/L and 67 U/L, respectively. Subsequently, they gradually began to increase. The serum amylase levels did not exceed the upper limit of the reference range after surgery. One and a half months postoperatively, the patient’s abdominal pain and diarrhea worsened, and she was readmitted to the hospital. Tenderness was observed from the middle to the left side of the lower abdomen, with no peritoneal irritation. Blood test results showed no increases in white blood cells or CRP, and the serum amylase level was normal at 48 U/L. Deviant hepatic enzymes showed mild exacerbation of AST (78 U/L) and ALT (153 U/L), and the patient’s total cholesterol and body weight were the lowest at 94 mg/dL and 25.5 kg, respectively, on readmission. Abdominal radiography showed no niveau, and abdominal echography showed a mottled fatty liver (Fig. 5) and slight intrahepatic bile duct dilation with no dilation of the main pancreatic duct.

Based on these findings, the patient was diagnosed with fatty diarrhea, intestinal peristaltic pain, and NAFLD associated with pancreatic exocrine dysfunction without postoperative pancreatic duct passage obstruction. To supplement pancreatic digestive enzymes, pancrelipase containing high-titer amylase, lipase, and protease was started at 900 mg/day divided between three meals. Although abdominal pain was alleviated by relieving diarrheal stools, steatorrhea persisted. Therefore, the dose of pancrelipase was gradually increased, and antidiarrheal agents (albumin tannate and natural aluminum silicate) were added.

The dose of pancrelipase was increased to 2250 mg daily divided between meals, and by using it in combination with an antidiarrheal agent, abdominal pain was almost alleviated except for defecation. The patient was discharged because her feculence improved from muddy to loose stools. During follow-up at an outpatient clinic, abdominal pain and fat diarrhea stools recurred when food or fat intake increased.

Including dietary guidance, the dose of pancrelipase was increased to 2700 mg/day, and albumin tannate and natural aluminum silicate were increased to 4.5 g divided between meals. Since muddy stools were still frequent, loperamide hydrochloride was added a dose of 0.5 mg/day. However, natural aluminum silicate was discontinued to avoid the stools from becoming too hard, and albumin tannate was reduced to a dose of 1.5 g/day after lunch. Upon reducing the dose and taking 0.6 mg of loperamide hydrochloride after breakfast and dinner, loose stools could be relieved approximately two or three times a day. However, stools may change from muddy to loose stools, depending on the content and amount of food.

At the time of writing, one year and two months have passed since the operation. The patient’s height increased by 6 cm approximately one year postoperatively; she weighed 30.5 kg, and recovering from 25.5 kg at postoperative readmission to her preoperative weight. The patient currently attends school and after school lessons. Blood tests showed that AST remained at 50–60 U/L, ALT remained at 70–80 U/L, and total cholesterol level recovered to 147 mg/dL. The postoperative zinc levels have been within normal range. Although fatty liver remains to be observed on echography, no recurrence or metastasis of the pancreatic SPN was observed on CT.

## Discussion

Pediatric pancreatic tumors are extremely rare. However, pancreatic SPNs are the most common pancreatic tumors in children, with approximately 20% cases occurring in children [2]. Generally, PD is a surgical procedure that is rarely performed in children due to the rarity of diseases with lesions that might occur in their pancreatic heads. According to a National Clinical Database (NCD) report in the field of pediatric surgery in Japan, pancreatectomy is extremely rare, with 12 and 19 cases reported in 2017 and 2018, respectively [3]. Regarding pancreatic SPN, in pediatric patients (under 15 years of age), the site of occurrence was the pancreatic head (41.5%), body (34.0%), and tail (24.5%), and PD was performed in 6/22 (27.3%) pancreatic heads [4]. Therefore, there is little evidence of postoperative management after PD in children in the acute phase and medium-to-long term phases.

Pancreatic resection and decreased function of the residual pancreas are associated with NAFLD development after PD. However, as far as the literature could show, there is no data available on NAFLD development in children after PD. The main characteristic of this NAFLD is that, unlike ordinary NAFLD, it occurs after pancreatic resection, with worsening nutritional status, without insulin resistance. Although the mechanism is not well understood, some assumptions have been proposed [5–7].

One possible mechanism [5] is that malnutrition due to decreased pancreatic exocrine function might promote conversion of carbohydrates into fats in the liver. The main factors are neurogenic diarrhea associated with SMA plexus dissection, and impaired fat absorption and steatorrhea due to decreased pancreatic exocrine function. This impaired fat absorption is thought to increase carbohydrates conversion to fat in the liver.

Another possible mechanism [6] is that endotoxins may induce liver damage. Intractable diarrhea caused by dissection of the surrounding plexus or decreased pancreatic exocrine function may induce bacterial translocation due to intestinal mucosal atrophy. This may lead to endotoxin influx into the liver via the portal vein thereby activating Kupffer cells and causing fatty deposits in the liver. It has also been proposed that this mechanism may be related to a deficiency in the trace element of zinc [7]. A marked decrease in blood-zinc and zinc content in the pancreatic tissue has been reported after wide-area resection of the pancreas. Zinc is mostly absorbed in the duodenum and proximal jejunum by zinc-binding proteins in the pancreatic juice and plays an important role in the regeneration and maintenance of the structure of the intestinal mucosal epithelium. In addition to removing a part of the intestinal tract in PD, the amount of zinc isolated from food is also reduced due to decreased pancreatic exocrine function. In addition, post-PD insulin hypofunction increases urinary excretion of zinc, resulting in zinc deficiency. This is thought to decrease the protective effect of zinc on the intestinal mucosa, leading to increases in intestinal permeability and an influx of endotoxins through the mucosa.

Our patient was a 10-year-old girl who was not diagnosed of pancreatitis. In addition, no postoperative increases in serum amylase levels, dilation of the pancreatic duct, or pancreatic atrophy were observed. Therefore, we did not consider that PD would lead to postoperative exocrine pancreatic dysfunction. Postoperative pancreatic endocrine function could maintain the fasting blood glucose within the normal range (97 mg/dL) and HbA1c (5.7%).

In our case, the patient was noticeably emaciated preoperatively and underwent a partial resection of the SMA plexus. Postoperatively, watery diarrhea was observed first, followed by fatty diarrhea. Therefore, impaired fat absorption through the first mechanism is thought to enhance carbohydrates conversion to fat in the liver. Zinc deficiency was observed in the blood test results.

After PD, NAFLD has been reported to occur in 20–40% of cases [5, 8, 9]. It has been shown that pancreatic enzyme supplementation could improve fatty liver and liver damage in patients with NAFLD and ameliorate low nutrition, fatigue, and possible diarrhea. Kato et al. [5] reported that pancreatic enzyme (pancreatin) supplementation in patients with post-PD NAFLD resulted in a higher NAFLD remission rate in the high-dose group than in the normal-dose group. Furthermore, Nagai et al. [10] conducted a prospective study of patients with NAFLD after PD and reported that pancrelipase, an enteric-soluble granule of high-titer pancreatin, improved NAFLD assessed by CT and blood tests. Diarrhea symptoms also improved, suggesting that postoperative indigestion was the main cause of NAFLD. Therefore, pancrelipase was administered to self-test cases. Initially, we started with 900 mg/day, which is half of the normal dose in adults, considering body weight. However, the dose was temporarily increased to 2700 mg /day of pancrelipase due to continued steatodiarrhea. The combination of antidiarrheal agents (albumin tannate, natural aluminum silicate, and loperamide hydrochloride) resulted in tangible soft stools and reduced stool frequency. In addition, the patient in this case showed improvements in AST, ALT, total cholesterol, and weight gain.

## Conclusions

Our pediatric surgeons had little experience in PD in children and considered that they had sufficient residual pancreatic function, unlike in adult cases. However, we experienced a case of NAFLD after PD for pediatric pancreatic SPNs. Pancreatic enzyme supplementation was effective in improving this condition. In addition, since NAFLD may continue to worsen and progress to nonalcoholic steatohepatitis and even liver failure, it is important to continue pancreatic enzyme supplementation therapy and careful follow-up.

## Data Availability

Not applicable.

## Abbreviations

ALT: Alanine aminotransferase
AST: Aspartate aminotransferase
SPN: Solid pseudopapillary neoplasm
PD: Pancreatoduodenectomy
CT: Computed tomography
MRI: Magnetic resonance imaging
NAFLD: Nonalcoholic fatty liver disease
SMA: Superior mesenteric artery
NCD: National clinical database
